# Does botulinum toxin affect psycho-social aspects in dystonia?

**DOI:** 10.1007/s00702-024-02785-z

**Published:** 2024-06-04

**Authors:** Angelica Marfoli, Francesca Mameli, Edoardo Nicolò Aiello, Fabiana Ruggiero, Angelica De Sandi, Denise Mellace, Beatrice Curti, Roberto Vimercati, Barbara Poletti, Nicola Ticozzi, Daniela Chieffo, Gabriella Santangelo, Sergio Barbieri, Alberto Priori, Roberta Ferrucci

**Affiliations:** 1 University of Milan, Milan, Italy; 2 Foundation IRCCS Ca’ Granda Ospedale Maggiore Policlinico, Milan, Italy; 3 IRCCS Istituto Auxologico Italiano, Milan, Italy; 4 Catholic University of the Sacred Heart, Rome, Italy; 5 Luigi Vanvitelli University of Campania, Caserta, Italy; 6 ASST Santi Paolo e Carlo, University Research Centre Aldo Ravelli, Milan, Italy

**Keywords:** Focal dystonia, Botulinum toxin, Psycho-social correlates, Body image, Depression

## Abstract

Dystonia is a movement disorder in which sustained muscle contractions give rise to abnormal postures or involuntary movements. It is a disabling and disfiguring disorder that affects activities of daily living and gives people a bizarre appearance often associated with psychological morbidity, embarrassment and social avoidance. Intramuscular injection of botulinum toxin (BoNT) is the most effective treatment for motor symptoms in focal dystonia, but little is known about its impact on the psycho-social dimension. The main aim of this study was to evaluate psycho-social changes in patients with focal dystonia after starting BoNT treatment using self-reported scales. The Beck Depression Inventory (BDI-II), the 36-Item Short Form Health Survey (SF-36), the Body Uneasiness Test (BUT), the State-Trait Anxiety Inventory (STAI) and the Visual Analogue Scale (VAS) assessing body self-image, satisfaction with physical aspects, social avoidance, self-reported depression, and self-distress were completed by 11 patients with dystonia and 9 patients with hyperhidrosis as a control group before BoNT (T0). VAS was then performed after four weeks (T1) to assess whether BoNT induced changes in the psychosocial dimension. Our results showed that only depressive symptoms and rumination about body defects improved in patients with dystonia after BoNT treatment, while improvement in self-distress and satisfaction with physical aspects was also found in hyperhidrosis. Individuals with hyperhidrosis experience poorer psychological well-being and suffer from higher levels of distress compared to dystonic patients. This suggests that individuals with this disabling condition are more vulnerable to social impact than dystonic patients.

**Supplementary Information:**

The online version contains supplementary material available at 10.1007/s00702-024-02785-z.

## Introduction

Dystonia is a movement disorder characterized by heterogenous sustained or intermittent muscle contractions leading to abnormal postures or involuntary movements that are typically patterned and twisted and often accompanied by tremors. Dystonia is often induced or exacerbated by voluntary actions related to excessive muscle activation (Davidescu et al. 2021). Originally, dystonia was classified as basal ganglia (BG) disorder, with abnormalities in the dopaminergic activity of BG accused for the disease (Degirmenci et al. 2013). However, although it is now regarded as a “*network*” disorder, including the cerebellum, the certain pathogenesis remains unkown (Kaji et al. 2018). In fact, based on limited data, there is evidence that pathophysiology of dystonia may involve a combination of dysfunction within neurons of the brainstem, cerebellum, putamen, and globus pallidus (Sharma 2019), with functional disorganization being reported in patients’somatosensory cortex (Kaji et al. 2018).

Currently, dystonia is classified according to two axes. The first axis categories dystonia on the basis of age at onset, body distribution and temporal pattern, while the second etiologic axis includes inheritance patterns, mode of acquisition, or unknown causality (Grutz & Klein, 2021).

The present study focuses on focal dystonia, in which a single region of the body is affected, specifically on cervical dystonia or torticollis (neck), hemispasm (face), and blepharospasm (eyes) (Pekmezovic et al. 2009; Steeves et al. 2012). Although focal dystonia has long been considered a primarily motor disorder, in recent decades increasing research has shown that patients suffering from this condition also experience non-motor symptoms such as psychological and psychiatric problems resulting from the disease and perceived social stigma among healthy individuals, which could negatively impact important aspects of the patient’s social and occupational activities of daily life and quality of life (QoL). In particular, there is evidence that this disabling and disfiguring disorder can give people a bizarre appearance associated with embarrassment, often leading to psychological morbidity, social avoidance and withdrawal. Several studies have also shown that patients with focal dystonia often have higher levels of anxiety and major depressive disorders compared to healthy individuals (Camargo et al. 2015; Degirmenci et al. 2013).

Intramuscular injection of botulinum toxin (BoNT), a neurotoxin that blocks the release of acetylcholine from the presynaptic terminal of neuromuscular junction (Huang et al. 2000), is currently considered the therapy of choice for motor symptoms in focal dystonia (Dressler et al. 2021; Camargo et al. 2015; Bledsoe et al. 2016; Colosimo et al. 2012; Muller et al. 2002; Cole et al. 1995), both with respect to the high toleration level for this substance as well as to the effective therapeutic impact generated (Dressler et al. 2021; Costanzo et al. 2021). In a recent study, Dressler and colleagues (2021) confirmed previous data concerning the effectiveness of botulinum injections into dystonic muscles in producing a well controllable peripheral paresis, with adverse effects appearing to be mild and transient.

However, to our knowledge and previous literature review, no studies have specifically examined the effects of BoNTs on psychosocial variables related to body self-image in patients with focal dystonia. For example, research in this area has recently shown that intramuscular botulinum toxin injections are effective in reducing psychological symptoms in terms of feelings of anxiety and depression in patients with dystonia (Costanzo et al. 2021), but both psychological stress and social consequences correlate with body self-image not yet investigated.

The main objective of the present work was to assess in what extent focal dystonia affects psychological and perceived health status and body image perception and satisfaction, with the specific aim of evaluating the possible effect of BoNT treatment on body self-image perception, self-reported depression, self-distress, satisfaction with physical aspects and social withdrawal. For this purpose, we tested psycho-social correlates at two different timepoints – before BoNT injection (T0) and four weeks after BoNT therapy (T1).

## Methods

### Study participants and ethics

Twenty-three patients were enrolled from among outpatients at the Movement Disorders clinic of the Foundation IRCCS Ca’ Granda Ospedale Maggiore Policlinico of Milan, Italy. The entire sample (*n* = 20) included patients diagnosed with focal dystonia (*n* = 11; 67.9 ± 8.8) according to validated criteria, specifically cervical dystonia (*n* = 2), blepharospasm (*n* = 6), and hemispams (*n* = 3), and patients diagnosed with primary focal hyperidrosis (*n* = 9; 39.8 ± 10.6) as control group. Hyperhidrosis is a disorder characterized by excessive sweating in specific areas of the body, for example, hands’ palms, the soles of the feet or the armpits, which causes discomfort, embarrassment and may lead to serious psychological, social and occupational impairments. Exclusion criteria were generalized and secondary forms of dystonia. Demographic and clinical data, including age, educational level, disease duration and gender were collected (Table 1). The experimental procedure was approved by the local institutional review board (Foundation IRCCS Ca’ Granda Ethics Committee) and was conducted in accordance with the Declaration of Helsinki. All patients were informed of the purpose of the study and written informed consent was obtained from all participants.

### Psycho-social correlates assessment

All patients underwent one evaluation of psycho-social correlates before botulinum toxin treatment (baseline) by performing the following tests: the Beck Depression Inventory II (BDI-II), the 36-Item Short Form Health Survey (SF-36), the Body Uneasiness Test (BUT), the State-Trait Anxiety Inventory (STAI) and the Visual Analogue Scale (VAS). After four weeks from botulinum toxin injection only VAS was administrated by telephone in order to assess whether BoNT therapy induced changes on psychological and social symptoms in patients diagnosed with focal dystonia.

### Assessment scales

The *Beck Depression Inventory II* (BDI-II) (Beck et al. 1996) was performed to evaluate the presence and severity of depression. It is a self-report questionnaire consisting of 21 items ranging from 0 to 3. Higher total scores indicate more severe depressive symptoms. The cut-offs are the following: 0–3 for minimal depression; 14–19 for mild depression; 20–28 for moderate depression; 29–63 for severe depression.

The *36-Item Short Form Health Survey* (SF-36) (Brazier et al. 1992) is an instrument composed of 36 questions assessing the quality of life (QoL) and the general state of health. The reference values range from 0 (total health impairment) to 100 (total well-being state). The assessed domains are: Physical Functioning (PF); Role-Physical (RP); Bodily Pain (BD); General Health (GH); Vitality (V); Social Functioning (SF); Emotional Role (ER); Mental Health (MH).

The *Body Uneasiness Test* (BUT) (Cuzzolaro et al. 2006) was used to evaluate body dissatisfaction index and the presence of depersonalization and derealization symptoms towards one’s body. This self-rated questionnaire includes 34 items whose scores on a Liker Scale range from 0 (never) to 5 (always). It provides several scores or index: in addition to the total scores, the *Global Severity Index* (GSI) identifies the degree of severity correlated to one’s body self-image. It includes 5 subscales or factors: Weight Phobia (WP); Body Image Concerns (BIC), Compulsive Self-Monitoring (CSM); Avoidance (A); Depersonalization (D).

The *State Trait Anxiety Inventory* (STAI) (Spielberger et al. 1970) is composed of 40 items, 20 of which assess state anxiety and trait anxiety. State anxiety refers to an emotional state at a given moment, while trait anxiety is referred to a personological characteristics. Specifically, this instrument is composed of 20 questions with scores ranging from 1 to 4 for each item (almost never, sometimes, often, always) (Spielberger 1994). The final total score varies from 20 to 80: 80 − 71 for very high level; 70 − 51 for medium-high level; 50 − 31 for medium-low level; 30 − 20 for very low level or none.

The *Visual Analogue Scale* (VAS) is an instrument in which 11 items were processed for the assessment of subjective variations in worry and satisfaction with body image aspects and self-distress related to disease. It consists of a line usually 100 mm in length in which 0 indicates the minimum and 10 indicates the maximum, and the patient makes a mark reflecting its own perception.

### Statistics

#### Background analyses

Due to the restricted sample size, baseline between-group comparisons were run *via* Mann-Whitney’s tests. Similarly, the association between psychological measures at T0 and the change in each VAS item (computed as the raw difference between T1 and T0 scores; VASΔ) were explored, separately for the two groups, by means of Bonferroni-corrected Spearman’s correlations.

#### Effects of BoNT treatment on VAS scores

VAS − 3 and − 10 scores were reversed since, at variance with remaining ones, these items were negatively oriented.

Data distribution was then checked on raw VAS scores both by assessing skewness and kurtosis values (judged as abnormal if >|1| and >|3|, respectively [Kim 2013]) and by visually inspecting histograms and Q-Q plots. With the exception of VAS items 10 and 11, which distributed Normally, remaining ones were heavily right-skewed and overdispersed. Accordingly, linear models were employed when addressing VAS items 10 and 11, whilst generalized linear models underlying a Negative Binomial (Green 2021) distribution were employed for VAS items 1 to 9.

In order to test the effects of *Time*, *Group* and their interaction, linear/generalized linear mixed models were run separately on each VAS item by addressing *Subject* as the cluster, *Time* as the within-subject factor and *Group* as the between-subject factor. A random intercept was fitted within the *Subject* cluster. Bonferroni-corrected post-hoc comparisons were run for significant terms.

Analyses were run *via* IBM® SPSS® Statistics 29 (IBM Corp. 2023) and Jamovi 2.3 (The Jamovi Project 2022). Significance thresholds were Bonferroni-corrected whenever appropriate.

## Results

### Background analyses

Table 1 summarizes both group’s background and clinical measures at baseline. The two groups were matched for the vast majority of variables with the exception of age (dystonia patients being older than hyperhidrosis ones), education (being higher in hyperhidrosis patients when compared to dystonia ones) as well as BUT-PST and BUT-CSM scores (both being higher in hyperhidrosis patients than dystonia ones).

Supplementary Table 1 shows Spearman’s correlation coefficients between BDI-II, STAI, SF-36 and BUT scores and VASΔ scores. Overall, in both groups, no associations survived Bonferroni’s correction (α_adjusted_ = 0.005), with the exception of those between VASΔ-3 and SF-36-GH scores (*r*_*s*_(9)=-0.88; *p* = .002), as well as between both VASΔ-8 and VASΔ-9 and BUT-Avoidance scores (VASΔ-8: *r*_*s*_(9)=-0.92; *p* = .001; VASΔ-9: *r*_*s*_(9)=-0.90; *p* = .001) in hyperhidrosis patients.

**Table 1:** Patients’ background and clinical measures at baseline

	Dystonia	Hyperhidrosis	p*
N	11	9	
Age (months)	67.9 ± 8.8 (48–81)	39.8 ± 10.6 (20–48)	< 0.001
Sex (male/female)	45.5%/54.5%	33.3%/66.7%	0.582
Education (years)	9.9 ± 3.5 (5–13)	14.1 ± 1.8 (13–17)	0.005
Disease duration (years)	18.4 ± 5.7 (10–25)	24.8 ± 11.4 (5–40)	0.175
Phenotypes (%)			
Blepharospasm	54.5%	-	
Cervical dystonia	18.2%	-	
Hemispasm	27.3%	-	
BDI-II	5.3 ± 5.3 (0–17)	2.6 ± 4.2 (0–12)	0.089
STAI	32.5 ± 9.6 (21–46)	36 ± 11.2 (23–54)	0.402
SF-36			
PF	88.6 ± 12.3 (70–100)	98.3 ± 3.5 (90–100)	0.071
RP	88.6 ± 25.9 (25–100)	91.7 ± 25 (25–100)	0.760
BP	75.1 ± 23.6 (41–100)	93.3 ± 10.6 (72–100)	0.080
GH	80 ± 15.4 (50–100)	82.2 ± 11.4 (61–97)	0.969
V	79.1 ± 13.8 (50–100)	71.1 ± 9.6 (50–80)	0.132
SF	87.4 ± 13.8 (62–100)	87.3 ± 16.7 (62–100)	0.935
RE	94 ± 20.2 (33–100)	88.9 ± 33.3 (0-100)	0.884
MH	81.1 ± 17.3 (48–100)	76.9 ± 12 (56–92)	0.467
BUT			
GSI	0.3 ± 0.4 (0-1.15)	0.6 ± 0.4 (0.029-1)	0.128
PST	6 ± 7 (0–18)	13.2 ± 7.6 (1–23)	0.043
PSDI	1.2 ± 0.7 (0-2.33)	1.3 ± 0.3 (1-1.86)	0.586
BIC	0.4 ± 0.5 (0-1.33)	0.7 ± 0.5 (0-1.44)	0.125
A	0.1 ± 0.2 (0-0.5)	0.2 ± 0.4 (0-1.17)	0.708
CSM	0.2 ± 0.3 (0-0.83)	0.6 ± 0.3 (0.167-1)	0.005
D	0.1 ± 0.1 (0-0.2)	0.1 ± 0.2 (0-0.4)	0.321

*Notes* *p-values are associated with Mann-Whitney *U*-statistics, except for the comparison addressing sex, where it refers to a χ^2^-statistic for independent samples. BDI = Beck Depression Inventory; STAI-Y2 = State- and Trait-Anxiety Inventory-Form Y – Trait-Anxiety; SF-36 = Short-Form Health Survey; PF = Physical Functioning; RP = Role Physical; BP = Bodily Pain; GH = General Health; V = Vitality; SF = Social Functioning; RE = Role Emotional; MH = Mental Health; BUT = Body Uneasiness Test; GSI = Global Severity Index; PST = Positive Symptom Total; PSDI = Positive Symptom Distress Index; BIC = Body Image Concerns; A = Avoidance; CSM = Compulsive Self-Monitoring; D = Depersonalization

### Effects of BoNT treatment on VAS scores

Table 2 shows both groups’ VAS scores at the two time-points, whilst Table 3 reports the results of each model run.

A significant main effect of *Time* was detected on VAS-1 scores, with scores on this measure being overall lower at T1 (*M* = 0.47; *SE* = 0.19) when compared to T0 (*M* = 0.89; *SE* = 0.33) net of group belonging.

**Fig. 1:**
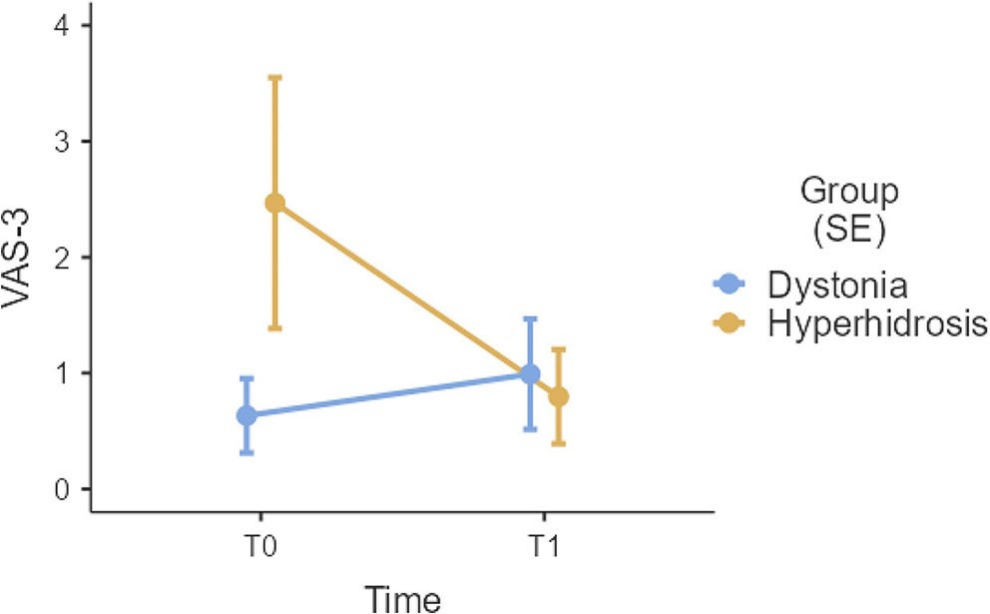
Visual display of the significant Time*Group interaction on VAS-3 scores. Represented values are estimated marginal means ± SE. VAS-3 = Visual Analogue Scale-“I am satisfied with my physical aspect”

A significant Time*Group term yielded with regard to VAS-3 scores (Fig. 1), whose post-hoc decomposition revealed that such a significance was selectively driven by the fact that hyperhidrosis patients (*p* = .012), but not dystonia ones (*p* = 1.000, reported significantly lower scores on this measure at T1 (*M* = 0.80; *SE* = 0.41) when compared to T0 (*M* = 2.47; *SE* = 1.08).

As to the VAS-4, a significant main effect of *Group* was detected, with hyperhidrosis patients reporting higher scores on this measure (*M* = 1.02; *SE* = 0.65) when compared to dystonia ones (*M* = 0.06; *SE* = 0.06) at both time-points.

A significant main effect of *Time* was detected on VAS-10 scores, with lower values being found at T1 (*M* = 1.73; *SE* = 0.41) when compared to T0 (*M* = 2.98; *SE* = 0.41) in both patient groups.

**Fig. 2:**
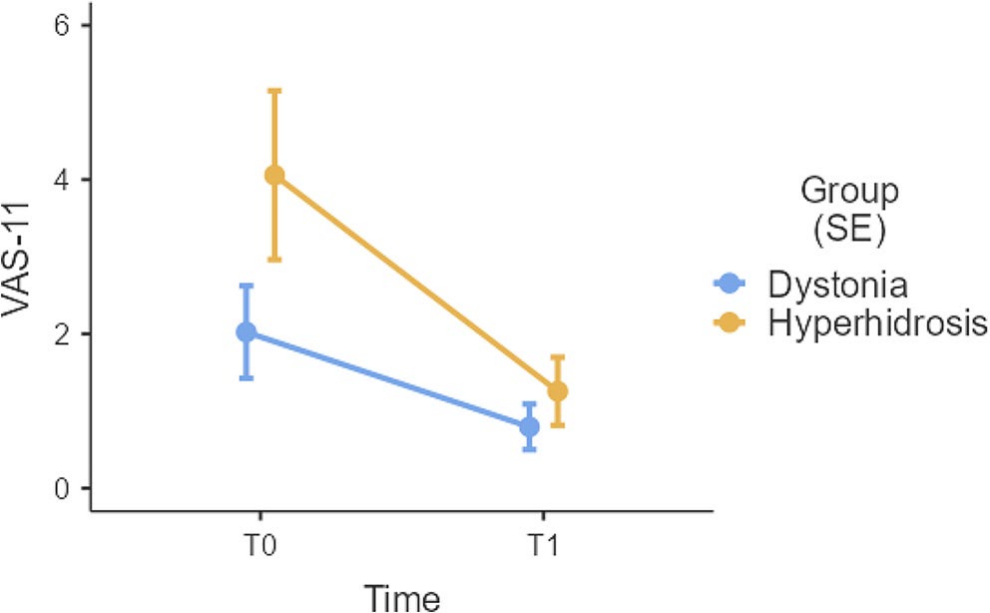
Visual display of the marginally significant Time*Group interaction on VAS-11 scores. Represented values are estimated marginal means ± *SE*. VAS-11 = Visual Analogue Scale-“Distress related to the disease”

Finally, the model on the VAS-11 yielded both a significant main effect of *Time* – with scores being overall lower at T1 (*M* = 1.22, *SE* = 0.44) when compared to T0 (*M* = 3.61, *SE* = 0.44) net of *Group*; however, when exploratively assessing a marginally significant Time*Group interaction term (Fig. 2), post-hoc comparisons revealed that such a decrease in scores was actually significant (*p* < .001) only for hyperhidrosis patients (T0: *M* = 4.67, *SE* = 0.65; T1: *M* = 1.44, *SE* = 0.65) and not for dystonia ones (*p* = .110).

**Table 2:** Patients’ VAS scores across the two time-points

	Dystonia		Hyperhidrosis	
	T0	T1	T0	T1
VAS-1	1.3 ± 1.7 (0–5)	1 ± 1.6 (0–5)	2 ± 2.4 (0–6)	0.8 ± 1.3 (0–4)
VAS-2	0.2 ± 0.6 (0–2)	0 ± 0 (0–0)	1.7 ± 2 (0–6)	0.4 ± 0.7 (0–2)
VAS-3	8.7 ± 2.2 (4–10)	8 ± 3.2 (0–10)	6.6 ± 2.8 (2–10)	8.9 ± 1.2 (7–10)
VAS-4	0.3 ± 0.6 (0–2)	0.1 ± 0.3 (0–1)	2.9 ± 2.7 (0–7)	1.9 ± 3.5 (0–10)
VAS-5	0.1 ± 0.3 (0–1)	0 ± 0 (0–0)	0.6 ± 1.3 (0–4)	0.1 ± 0.3 (0–1)
VAS-6	0.1 ± 0.3 (0–1)	0 ± 0 (0–0)	0.3 ± 0.7 (0–2)	0.1 ± 0.3 (0–1)
VAS-7	0.1 ± 0.3 (0–1)	0 ± 0 (0–0)	0.6 ± 1.1 (0–3)	0.1 ± 0.3 (0–1)
VAS-8	0.3 ± 0.6 (0–2)	0 ± 0 (0–0)	1.8 ± 3 (0–7)	0.8 ± 1.7 (0–5)
VAS-9	0.7 ± 1.4 (0–4)	0.3 ± 0.6 (0–2)	1.3 ± 1.7 (0–4)	0.8 ± 1.2 (0–3)
VAS-10	7.3 ± 2.1 (3–10)	8.1 ± 1.4 (5–10)	6.8 ± 2.3 (3–10)	8.4 ± 1.2 (7–10)
VAS-11	2.6 ± 2.2 (0–5)	1 ± 1.5 (0–5)	4.7 ± 2.4 (0–8)	1.4 ± 1.6 (0–4)

*Notes* VAS **=** Visual Analogue Scale; 1= “I spent a lot of time thinking of defects of my body image”; 2= “I was worried that my body would suddenly change”; 3= “I am satisfied with my physical aspect”; 4= “I felt different from how others saw me”; 5= “The thought of my body’s defects has tormented me so much that I was prevented from staying with other; 6= “The thought of my body’s defects has tormented me so much that I was prevented from working”; 7= “The thought of my body’s defects has tormented me so much that I was prevented from sexuality”; 8= “I felt embarrassed for my body”; 9= “I spent a lot of time checking my body defect in the mirror”; 10= “My mood has been”; 11= “Distress related to the disease”

**Table 3:** Results of the models testing the effect of BT on VAS scores in the two groups

		F/χ^2^	*p*
VAS-1			
	*Time*	**4.54**	0.033
	*Group*	0.24	0.627
	Time*Group	1.08	0.298
VAS-2			
	*Time*	0.11	0.737
	*Group*	0.15	0.701
	Time*Group	0.06	0.784
VAS-3			
	*Time*	1.82	0.177
	*Group*	0.88	0.35
	Time*Group	**9.80**	0.002
VAS-4			
	*Time*	1.65	0.199
	*Group*	**7.51**	0.006
	Time*Group	0.30	0.584
VAS-5			
	*Time*	0.05	0.827
	*Group*	0.05	0.826
	Time*Group	0.03	0.859
VAS-6			
	*Time*	0.00	0.975
	*Group*	0.00	0.974
	Time*Group	0.00	0.976
VAS-7			
	*Time*	0.04	0.845
	*Group*	0.04	0.842
	Time*Group	0.03	0.874
VAS-8			
	*Time*	0.17	0.681
	*Group*	0.21	0.645
	Time*Group	0.14	0.712
VAS-9			
	*Time*	3.51	0.061
	*Group*	1.22	0.270
	Time*Group	0.33	0.566
VAS-10			
	*Time*	**8.93**	0.008
	*Group*	0.01	0.921
	Time*Group	1.04	0.321
VAS-11			
	*Time*	**28.82**	< 0.001
	*Group*	2.88	0.107
	Time*Group	3.56	0.075

***Notes*** VAS **=** Visual Analogue Scale; 1 = VAS **=** Visual Analogue Scale; 1= “I spent a lot of time thinking of defects of my body image”; 2= “I was worried that my body would suddenly change”; 3= “I am satisfied with my physical aspect”; 4= “I felt different from how others saw me”; 5= “The thought of my body’s defects has tormented me so much that I was prevented from staying with other; 6= “The thought of my body’s defects has tormented me so much that I was prevented from working”; 7= “The thought of my body’s defects has tormented me so much that I was prevented from sexuality”; 8= “I felt embarrassed for my body”; 9= “I spent a lot of time checking my body defect in the mirror”; 10= “My mood has been”; 11= “Distress related to the disease”

## Discussion

The aim of the present study was to evaluate whether botulinum toxin therapy is effective in producing psycho-social and mood changes in a cohort of patients diagnosed with focal dystonia, in particular cervical dystonia, blepharospasm and hemispasm. We expected that individuals with this movement disorder would have shown improvement in these areas compared to patients with hyperhidrosis. Specifically, we longitudinally assessed dystonic and hyperhidrosis patients in order to detect possible modifications in both groups in relation to psychological and social aspects such as concerns and rumination on body self-image, body image satisfaction, body dysmorphic concerns, feelings of embarrassment for physical appearance, as well as social avoidance in interpersonal, occupational and sexual dimensions. In addition, a goal of this work was also to evaluate whether BoNT injections improve mood and reduce depressive symptoms and self-distress related to the disease in dystonia.

Overall, in our study we observed that before BoNT treatment hyperhidrosis patients experienced poorer psychological well-being and also suffered from higher levels of distress compared to dystonic patients, showing greater scores with regard to body dissatisfaction and more severe depersonalization and derealization symptoms. Furthermore, we also found that before BoNT therapy individuals with hyperhidrosis spent more time performing compulsive body image checks, and obsessively monitoring their physical aspects than dystonic ones. Therefore, these results suggest that patients with hyperhidrosis experience worse psychological status and greater dissatisfaction with body self-image before botulinum toxin injections.

With regard to botulinum toxin therapy, we observed that in both groups BoNT injections were associated with a reduction of ruminative thoughts, referring to the amount of time spent thinking of perceived defects in one’s own body image. Moreover, results also revealed that botulinum toxin therapy had a positive effect on mood tone, in terms of a decrease of depressive symptoms severity, with lower scores reported on VAS subitems. Other studies (Costanzo et al. 2021; Dong et al. 2018; Parsaik et al. 2016; Muller et al. 2002; Ochudlo et al. 2007; Degirmenci et al. 2013) demonstrated a reduction in depressive symptomatology following BoNT treatment in dystonia, possibly due to the symptomatic improvement in motor impairment. Moreover, Parsaik and colleagues (2016) in their systematic review and meta-analysis summarized data from further research evaluating the efficacy of botulinum toxin A on depression, whose analysis suggested that this protein’s injections produced significant improvement in depressive symptoms in patients suffering from several physical disorders.

However, even though in our study BoNT therapy has been shown to be effective in reducing self-reported depressive symptoms in both groups, we found that patients diagnosed with dystonia did not experience a reduction of distress correlated to the disease compared to hyperhidrosis group, who reported a significant improvement of psychological well-being associated with a reduction of self-distress. This result appears in line with data from recent studies evaluating the impact of hyperhidrosis on patients’ quality of life (QoL) (Almohideb et al. 2021; Henning et al. 2023; Parashar et al. 2023), and revealing that individuals suffering from this disease experience a reduced QoL which could lead to perceivable deteriorations in their mental wellness (Henning et al. 2013). From this perspective, the higher psychological well-being detected in these patients following BoNT therapy compared to dystonic ones may be correlated to the more severe impairment experienced in various functional domains as well as in the overall quality of life by individuals with hyperhidrosis due to their socially disabling disorder (Almohideb et al. 2021).

Moreover, contrary to our hypothesis, we did not find any effect of BoNT treatment on body self-image satisfaction in dystonic patients, while a decrease in body image dissatisfaction was found in patients diagnosed with hyperhidrosis. Again, these results could be explained by the poorer general psychological condition and more frequent body image compulsive checks observed in the latest group before botulinum toxin treatment in this study, indicating stronger psychological distress and concerns about physical appearance in hyperhidrosis compared to dystonia. In this sense, BoNT therapy may have had a greater action in reducing a more severe symptomatology in patients with hyperhidrosis compared to dystonic ones. At the same time, hyperhidrosis group exhibited higher feelings of being different from how others saw them both before and after BoNT injections compared to dystonia group, revealing that these patients, but not dystonic ones, continued to report body dysmorphia and altered body perceptions even after therapy. However, the increased responsiveness of individuals with hyperhidrosis to BoNT treatment may be related to the younger age of these patients as well as their higher level of education. According to the literature, younger people appear to be more concerned with their body image and physical aspects compared to the general population, which has a significant negative impact on their psychological well-being (Carvalho et al. 2020; Bergeron 2007). In addition, people with higher levels of education may have jobs where body image is more important, affecting their psychological well-being. For example, Smith and colleagues (2011) observed that participants with high status aspiration reported greater body dissatisfaction and ineffectiveness after being exposed to thin and successful woman than participants with low status aspiration. This information may suggest that younger people diagnosed with dystonia would have responded to BoNT therapy in the same way as patients with hyperhidrosis.

In conclusion, our findings suggest that BoNT treatment was effective in producing psychological variables changes as depressive symptoms reduction, and in decreasing the amount of time spent thinking of defects in one’s own body image in dystonia, while no associations were found between botulinum toxin therapy and the improvement of social aspects related to body self-image in the same group. However, contrary to expectations, patients with hyperhidrosis showed an improvement in body image satisfaction compared to dystonic patients and a reduction of self-distress related to illness, which may be related to a generally higher susceptibility to social consequences of their disease, suggesting that these individuals are more vulnerable to social aspects than dystonic patients.

### Limitations

The present study includes a small number of participants in both samples, which could limit the generalizability of the results obtained. Furthermore, we have not examined the extent of improvement in motor symptoms in dystonia and it is therefore not possible to determine with certainty whether improvement in depressive symptoms is accompanied by motor improvement or not.

### Electronic supplementary material

Below is the link to the electronic supplementary material.

Supplementary Material 1

## Data Availability

Datasets related to the present study are available upon reasonable request from interested researchers.
