# Supplementary material for: Does botulinum toxin affect psycho-social aspects in dystonia?
Source: J Neural Transm (Vienna). 2024 Jun 4;131(8):953–60. doi: 10.1007/s00702-024-02785-z (PMC11343871; doi:10.1007/s00702-024-02785-z)
Supplement: Supplementary file 1 — Supplementary Material 1 [file 702_2024_2785_MOESM1_ESM.docx]

**Supplementary Table 1.** Bonferroni-corrected Spearman’s coefficients between psychological outcomes and changes in VAS items in the two groups.

|  |  |  | **VASΔ-1** | **VASΔ-2** | **VASΔ-3** | **VASΔ-4** | **VASΔ-5** | **VASΔ-6** | **VASΔ-7** | **VASΔ-8** | **VASΔ-9** | **VASΔ-10** | **VASΔ-11** |
| --- | --- | --- | --- | --- | --- | --- | --- | --- | --- | --- | --- | --- | --- |
| **Dystonia** | **BDI** | | -0.33 | 0.50 | 0.17 | 0.50 | 0.50 | 0.50 | 0.50 | -0.07 | -0.28 | -0.48 | 0.17 |
|  | **STAI2** | | -0.50 | -0.10 | 0.51 | -0.10 | -0.10 | -0.10 | -0.10 | -0.43 | -0.52 | 0.07 | -0.01 |
|  | **SF-36** | |  |  |  |  |  |  |  |  |  |  |  |
|  | PF | | 0.47 | 0.47 | -0.41 | 0.47 | 0.47 | 0.47 | 0.47 | 0.53 | 0.29 | -0.21 | 0.15 |
|  | RP | | 0.47 | -0.15 | 0.03 | -0.15 | -0.15 | -0.15 | -0.15 | -0.22 | -0.17 | 0.03 | 0.17 |
|  | BP | | 0.49 | 0.00 | 0.23 | 0.00 | 0.00 | 0.00 | 0.00 | -0.08 | -0.12 | -0.13 | -0.10 |
|  | GH | | 0.55 | 0.05 | -0.29 | 0.05 | 0.05 | 0.05 | 0.05 | -0.09 | 0.06 | -0.21 | -0.08 |
|  | V | | 0.53 | -0.05 | -0.47 | -0.05 | -0.05 | -0.05 | -0.05 | 0.25 | 0.02 | -0.20 | 0.07 |
|  | SF | | 0.34 | 0.05 | -0.57 | 0.05 | 0.05 | 0.05 | 0.05 | 0.29 | 0.48 | -0.12 | 0.10 |
|  | RE | | .636 | -0.10 | -0.31 | -0.10 | -0.10 | -0.10 | -0.10 | -0.15 | -0.12 | 0.23 | 0.47 |
|  | MH | | 0.31 | 0.05 | -0.34 | 0.05 | 0.05 | 0.05 | 0.05 | 0.28 | 0.56 | 0.05 | -0.11 |
|  | **BUT** | |  |  |  |  |  |  |  |  |  |  |  |
|  | GSI | | -0.42 | -0.41 | .607 | -0.41 | -0.41 | -0.41 | -0.41 | -0.48 | -0.19 | 0.51 | -0.15 |
|  | PST | | -0.40 | -0.41 | .635 | -0.41 | -0.41 | -0.41 | -0.41 | -0.48 | -0.17 | 0.54 | -0.19 |
|  | PSDI | | -0.39 | -0.31 | 0.41 | -0.31 | -0.31 | -0.31 | -0.31 | -0.33 | -0.19 | 0.31 | 0.10 |
|  | BIC | | -0.37 | -0.51 | .665 | -0.51 | -0.51 | -0.51 | -0.51 | -0.59 | -0.39 | 0.44 | -0.14 |
|  | A | | -.754 | 0.19 | 0.32 | 0.19 | 0.19 | 0.19 | 0.19 | -0.18 | -0.02 | 0.04 | -0.06 |
|  | CSM | | -0.40 | -0.55 | 0.46 | -0.55 | -0.55 | -0.55 | -0.55 | -0.55 | -0.33 | 0.26 | -0.06 |
|  | D | | -.698 | -0.52 | 0.37 | -0.52 | -0.52 | -0.52 | -0.52 | -0.19 | -0.15 | 0.49 | -0.27 |
| **Hyperhidrosis** | **BDI** | | -0.56 | -0.43 | 0.63 | -0.32 | -0.10 | -0.10 | -0.03 | -0.35 | -0.29 | .802 | -0.65 |
|  | **STAI2** | | -.761 | -0.36 | 0.51 | -0.20 | 0.23 | 0.23 | 0.21 | -0.19 | -0.11 | 0.24 | -0.45 |
|  | **SF-36** | |  |  |  |  |  |  |  |  |  |  |  |
|  | PF | | 0.11 | -0.12 | 0.00 | -0.62 | -0.12 | -0.12 | -0.12 | 0.40 | 0.28 | -0.23 | 0.40 |
|  | RP | | 0.56 | 0.58 | -0.57 | 0.57 | -0.08 | -0.08 | -0.08 | -0.15 | -0.15 | -0.56 | 0.56 |
|  | DP | | 0.12 | 0.04 | -0.04 | 0.42 | -0.16 | -0.16 | -0.16 | -0.30 | -0.30 | -0.45 | 0.65 |
|  | GH | | .786 | .752 | **-.875** | 0.41 | 0.04 | 0.04 | 0.00 | 0.21 | 0.19 | -0.42 | 0.09 |
|  | V | | 0.60 | 0.11 | -0.14 | 0.31 | -0.28 | -0.28 | -0.19 | -0.09 | -0.14 | -0.09 | 0.56 |
|  | SF | | 0.42 | 0.02 | 0.00 | 0.53 | -0.12 | -0.12 | -0.03 | -0.26 | -0.23 | 0.00 | 0.36 |
|  | RE | | 0.56 | 0.58 | -0.57 | 0.57 | -0.08 | -0.08 | -0.08 | -0.15 | -0.15 | -0.56 | 0.56 |
|  | MH | | .706 | 0.20 | -0.32 | 0.22 | -0.37 | -0.37 | -0.33 | 0.00 | -0.08 | -0.19 | 0.58 |
|  | **BUT** | |  |  |  |  |  |  |  |  |  |  |  |
|  | GSI | | -0.42 | -0.35 | 0.48 | -0.04 | -0.56 | -0.56 | -0.54 | -0.63 | -.671 | 0.25 | 0.29 |
|  | PST | | -0.57 | -0.36 | 0.56 | -0.13 | -0.46 | -0.46 | -0.43 | -0.60 | -0.62 | 0.25 | 0.26 |
|  | PSDI | | -0.08 | -0.65 | 0.49 | -0.26 | -0.04 | -0.04 | -0.02 | 0.07 | 0.02 | -0.05 | 0.32 |
|  | BIC | | -0.36 | -0.39 | 0.44 | -0.07 | -0.59 | -0.59 | -0.59 | -0.57 | -0.63 | 0.24 | 0.27 |
|  | A | | -0.50 | -0.03 | 0.33 | 0.28 | -0.61 | -0.61 | -0.58 | **-.916** | **-.897** | 0.32 | 0.17 |
|  | CSM | | -0.32 | -0.49 | 0.55 | -0.27 | -0.14 | -0.14 | -0.09 | -0.19 | -0.22 | 0.03 | 0.37 |
|  | D | | -.743 | -0.32 | 0.63 | -0.06 | -0.61 | -0.61 | -0.57 | -.832 | -.825 | 0.62 | -0.03 |

**Notes.** Significant coefficients at the Bonferroni-corrected threshold of α_adjusted_=0.005 are in bold. BDI=Beck Depression Inventory; STAI-Y2=State- and Trait-Anxiety Inventory-Form Y – Trait-Anxiety; SF-36=Short-Form Health Survey; PF=Physical Functioning; RP=Role Physical; BP=Bodily Pain; GH=General Health; V=Vitality; SF=Social Functioning; RE=Role Emotional; MH=Mental Health; BUT=Body Uneasiness Test; GSI=Global Severity Index; PST=Positive Symptom Total; PSDI=Positive Symptom Distress Index; BIC=Body Image Concerns; A=Avoidance; CSM=Compulsive Self-Monitoring; D=Depersonalization.
